# Heterologous Expression and Antimicrobial Targets of a Novel Glycine-Rich Antimicrobial Peptide from *Artemia franciscana*

**DOI:** 10.3390/md23080330

**Published:** 2025-08-17

**Authors:** Ming Tao, Aobo Sun, Huishi Shao, Huaiyuan Ye, Guangming Yu, Daigeng Chen, Wei Zhang

**Affiliations:** 1 Biosafety Level 3 Laboratory of Shenzhen University, Shenzhen 518060, China; tsmniu@szu.edu.cn; 2 College of Life Sciences and Oceanography, Shenzhen University, Shenzhen 518060, China; 3 College of Marine Science BGU, Beibu Gulf University, Qinzhou 535011, China

**Keywords:** antimicrobial peptides, glycine-rich, antibacterial targets, *Artemia franciscana*

## Abstract

The growing problem of antimicrobial resistance in aquaculture, caused by the excessive and unregulated use of antibiotics, highlights the critical necessity for developing new anti-infective solutions. Based on the characteristics of glycine-rich antimicrobial peptides (AMPs) and transcriptomic data, an antimicrobial peptide, namely *Af*Rgly1, was discovered in this study. Subsequently, the peptide was obtained through heterologous expression in *E. coli*, and its antibacterial spectrum was determined. Molecular dynamics simulation and molecular biology experiments were conducted to explore the antibacterial target of *Af*Rgly1. Results showed that the mRNA expression level of *Af*Rgly1 was significantly upregulated after *Vibrio alginolyticus* infection. *Af*Rgly1 has broad-spectrum antibacterial activity targeting on bacterial cell membrane, and it may also interact with bacterial DNA. *Af*Rgly1 displayed low selectivity for fish red blood cells. These results indicate that *Af*Rgly1 is an antimicrobial peptide with considerable potential for application in the development of therapeutic agents.

## 1. Introduction

Aquaculture serves as a vital source of animal protein for human consumption [1]. However, disease outbreaks present significant challenges to achieving sustainable and eco-friendly development in this sector. The intensive farming methods employed in aquaculture often heighten the environmental stress on aquatic organisms, making them more vulnerable to pathogenic infections [2]. While antimicrobial agents are widely employed for disease prevention and treatment, their excessive application in aquaculture systems may contribute to the emergence of drug-resistant bacterial strains and the spread of resistance genes [2,3]. This phenomenon potentially endangers both aquatic species and humans. In this context, antimicrobial peptides (AMPs), the evolutionarily conserved components of innate immunity, have emerged as promising therapeutic alternatives against drug-resistant pathogens [4].

Invertebrates have evolved sophisticated defense mechanisms against microbial pathogens, primarily through the biosynthesis of diverse AMPs. Extensive research over the last twenty years has identified numerous AMP families across various invertebrate taxa, encompassing arthropods (insects, arachnids, crustaceans, and chelicerates) and mollusks [5]. These bioactive molecules exhibit remarkable structural heterogeneity, which permits their systematic classification into three principal categories based on their conformational characteristics: (1) linear polypeptides that are capable of adopting amphipathic α-helical configurations with distinct hydrophobic domains; (2) cyclic peptides that are stabilized by intramolecular disulfide bridges facilitating β-sheet or mixed α-helix/β-sheet folding patterns; and (3) amino acid-enriched peptides that contain disproportionate high concentrations of specific residues, particularly proline or glycine [6,7,8]

Glycine-rich antimicrobial peptides (AMPs), exemplified by Adepantin-1 (engineered through sequence-based AMP-Designer algorithms), Acanthoscurrin-1 (isolated from *Acanthoscurria gomesiana*), and Hyastatin (derived from *Hyas araneus*), exhibit remarkable structural plasticity owing to their distinctive glycine-enriched sequences, which typically comprise over 20% of their amino acid composition [8,9,10]. Since glycine lacks a side chain (containing only a hydrogen atom as the β-carbon substituent), its steric hindrance is significantly reduced, thereby conferring high structural flexibility and conformational diversity [11,12]. Numerous glycine-rich AMPs contain repetitive sequences of (Gly-x)n motifs, with n varying in length; these sequences commonly adopt antiparallel β-sheet conformations or exhibit flexible coiled structures [13]. Leptoglycine (isolated from *Leptodactylus pentadactylus* skin secretion), Holotricin-3 (found in *Holotrichia diomphalia*), and Serrulin (found in *Tityus serrulatus*) are also typical members of glycine-rich AMPs with good antibacterial activity [14,15,16].

*Artemia franciscana* (brine shrimp) is a small arthropod that inhabits high saline environments such as salt lakes and salt pans [17]. Arthropods solely rely on their innate immunity to resist pathogenic microorganisms, thus becoming one of the most important sources of AMPs [18]. Some confirmed AMPs, such as Cecropin, Melittin, and Crustin, have all been discovered from arthropods [19,20,21]. However, AMPs in *A. franciscana* have been rarely reported. Based on the characteristics of glycine-rich AMPs and transcriptome data, a novel glycine-rich antimicrobial peptide (named *Af*Rgly1) was discovered from brine shrimp and heterologous produced. Experiments revealed that it has broad-spectrum antibacterial activity. The antibacterial target of *Af*Rgly1 was further analyzed through molecular dynamics simulation and molecular experiments. This research will serve as a novel template for the development of AMPs, offering valuable insights into their design and application.

## 2. Results

### 2.1. Screening of AMPs with Rich Glycine

**Figure 1:**
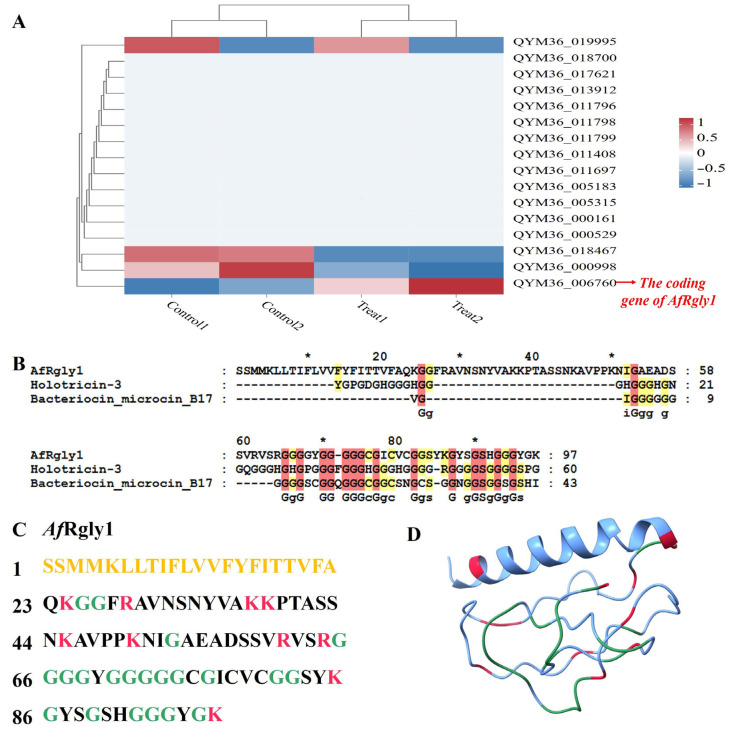
The response characteristics of glycine-rich antimicrobial peptide coding genes to *V. alginolyticu*s stress and sequence analysis of *Af*Rgly1. (**A**) The response characteristics of glycine-rich antimicrobial peptide coding genes to *V*. *alginolyticus* stress and sequence analysis of *Af*Rgly1. (**B**) The alignment results between AfRly1 and the two sequences most similar to it in the CAMPR3 database. (**C**) The amino acid sequence of *Af*Rgly1 is presented, with signal peptide residues indicated in yellow. Cationic amino acids, including lysine and arginine, are displayed in red, while glycine residues are marked in green. (**D**) The predicted three-dimensional structure of *Af*Rgly1, generated using AlphaFold3, cationic amino acids (lysine and arginine) are shown in red, and glycine residues are labeled in green. * indicates that this is the position of the 10th, 30th, 50th, 70th and 90th amino acids.

The protein profile derived from the *A*. *franciscana* genome revealed the existence of 26,923 predicted proteins [22]. With the fact that AMPs typically consist of fewer than 100 amino acids [23], 1718 predicted proteins shorter than 100 amino acids were screened out. Among them, there are 16 sequences containing more than 20% glycine (Supplementary Table S1) and with extracellular localization. After stimulation with *Vibrio alginolyticus*, only the gene with NCBI accession number QYM36_006760 was found to be significantly upregulated (Figure 1A and Supplementary Table S2), and it is the coding gene of *Af*Rgly1 (NCBI access number: KAK2718072.1). The sequence alignment analysis using the BLASTP program in the CAMPR3 database (accessed on 7 August 2025) revealed that *Af*Rgly1 is similar to Holotricin-3, with an identity of 38.09% (Figure 1B and Supplementary Table S3).

### 2.2. Sequence and Structure Characterization of AfRgly1

The amino acid sequence of *Af*Rgly1 contains 97 residues (Figure 1C), with a calculated molecular weight of 9.79 kDa and a protein isoelectric point value of 9.80 predicted by APD3 [24]. The Grand Average Hydropathy value of *Af*Rgly1 is 0.001, and the total net charge of *Af*Rgly1 is + 9.25. The CDS encoding *Af*Rgly1 is 291 bp in length. *Af*Rgly has an α-helix at its N-terminal, while the rest is linear (Figure 1D). WoLF PSORT predicted its localization as extracellular.

### 2.3. Recombinant Expression and Purification of AfRgly1

**Figure 2:**
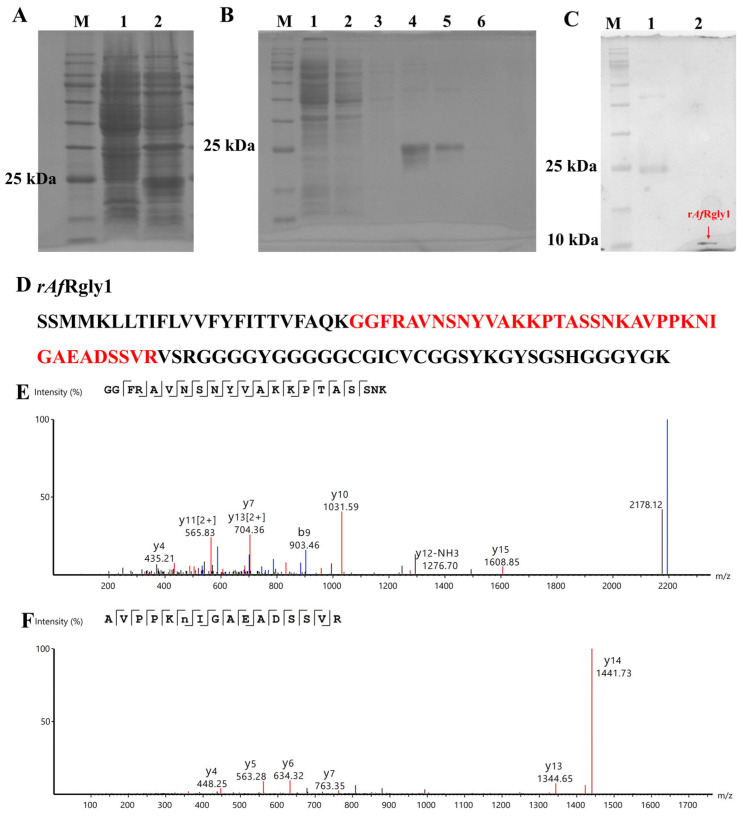
Acquisition process and MS spectrum analysis of r*Af*Rgly1. (**A**) SDS-PAGE analysis of recombinant *Af*Rgly1 (r*Af*Rgly1) fused with a His-SUMO tag in *E. coli*. Lane M, protein marker; lane 1, total protein obtained from *E. coli* without IPTG induction; lane 2, total protein obtained from *E. coli* with IPTG induction. (**B**) His-SUMO-*Af*Rgly1 was purified via nickel column chromatography. Lane M, protein marker; lane 1, protein not caught by the nickel column; lane 2, equilibration buffer; lane 3, eluent with 50 mM imidazole; lane 4, eluent with 100 mM imidazole; lane 5, eluent with 200 mM imidazole; lane 6, eluent with 300 mM imidazole. (**C**) SDS–PAGE analysis of r*Af*Rgly1 without the SUMO tag. Lane M, protein marker; lane 1, His-SUMO-*Af*Rgly1 before treatment with the SUMO enzyme; lane 2, red arrow points to the band of tag-free r*Af*Rgly1. (**D**) Alignment of mass spectrometry results with the r*Af*Rgly1 sequence. The red area compares mass spectrometry results with the r*Af*Rgly1 sequence. (**E**,**F**) MS spectra of “GGFRAVNSNYVAKKPTASSNK” and “AVPPKNIGAEADSSVR”, respectively.

Using pSmartI as the vector template, the mature peptide of *Af*Rgly1 was fused to the C-terminus of the His-SUMO tag. SDS-PAGE analysis revealed marked differences in the banding patterns, with approximately 25 kDa proteins detected in the bacteria after IPTG induction (Figure 2A). This specific band corresponds to the expected size of the His-SUMO-*Af*Rgly1 fusion protein, which consists of a His-SUMO tag (approximately 18 kDa) and *Af*Rgly1 (9.79 kDa). The complex of fusion protein His-SUMO-*Af*Rgly1 can be eluted from the nickel column via gradient elution with imidazole eluent at the optimal concentration of 200 μM (Figure 2B, lane 5). After treatment with the SUMO enzyme, recombinant *Af*Rgly1 (r*Af*Rgly1) without the His-SUMO tag (approximately 10 kDa) was obtained and confirmed by SDS-PAGE (Figure 2C).

### 2.4. Liquid Chromatography and Mass Spectrometry (LC-MS) Identification of AfRgly1

The amino acid sequence of r*Af*Rgly1 was analyzed by LC-MS. As shown in Figure 2E, two peptides were detected in total, whose coverage reached 38.14%.

### 2.5. Antimicrobial Activity of rAfRgly1

The antibacterial activity of r*Af*Rgly1 against three Gram-positive bacteria and four Gram-negative bacteria was determined by measuring the minimum inhibitory concentration (MIC). As shown in Table 1, r*Af*Rgly1 exhibited significant inhibitory activity against both Gram-positive and Gram-negative bacteria. For Gram-negative bacteria, 64 μM of r*Af*Rgly1 effectively inhibited the growth of *V*. *alginolyticus*, *Aeromonas hydrophila*, *Vibrio anguillarum,* and *Escherichia coli*. No growth inhibitory effect of r*Af*Rgly1 on *Acinetobacter* sp. L32 *and Vibrio harveyi* was observed at 64 μM. The MIC values of r*Af*Rgly1 against *Staphylococcus aureus*, *Bacillus* sp. T2 were lower, which was 32 μM. The antibacterial effect of r*Af*Rgly1 is not as good as that of r*Pp*Rcys1 in our previous study.

### 2.6. Molecular Dynamics (MD) Simulations of AfRgly1

**Figure 3:**
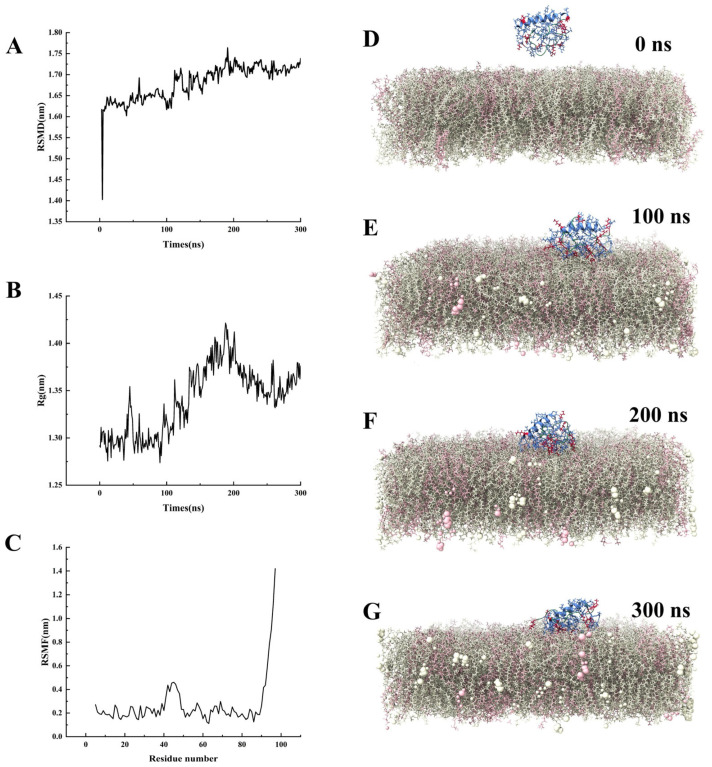
Molecular dynamics simulation of *Af*Rgly1 binding to membrane. (**A**) The RSMD of the *Af*Rgly1. (**B**) The Rg of the *Af*Rgly1. (**C**) The RSMF of the *Af*Rgly1. (**D**–**G**) Time-dependent membrane interaction behavior of AfRgly1 observed throughout the molecular dynamic simulations (0–300 ns). Cationic amino acids (lysine and arginine) are shown in red, and glycine residues are labeled in green.

Before 200 ns, the root mean square distances (RMSD) value continuously increased, indicating significant conformational changes in the protein (Figure 3A,D–F). After 200 ns, the fluctuation range of RMSD gradually decreased and stabilized, suggesting that the system reached a relatively stable conformational state (Figure 3A,F,G). At 50 ns and 200 ns, the radius of gyration (Rg) value suddenly increased and then decreased, indicating that the protein underwent local unfolding and refolding at these two time points (Figure 3B). The root mean square fluctuation (RMSF) values of residues SER41-LYS50 and SER90-LYS97 were relatively high, suggesting that these two regions exhibited strong dynamic fluctuations during the membrane contact process (Figure 3C). Molecular dynamics simulation snapshots showed that the *Af*Rgly1 protein exhibited dynamic behavior of actively approaching the membrane within 0–300 ns (Figure 3D–G). At 100 ns, two positively charged residues, LYS50 and LYS85, were the first to insert into the membrane interface (Figure 3D). At 300 ns, the number of inserted residues increased to four (LYS37, LYS45, LYS50, and LYS85), indicating that the interaction between the protein and the membrane was gradually strengthened over time (Figure 3G).

### 2.7. Microorganism-Binding and Membrane Mimetic-Binding Activity of rAfRgly1

**Figure 4:**
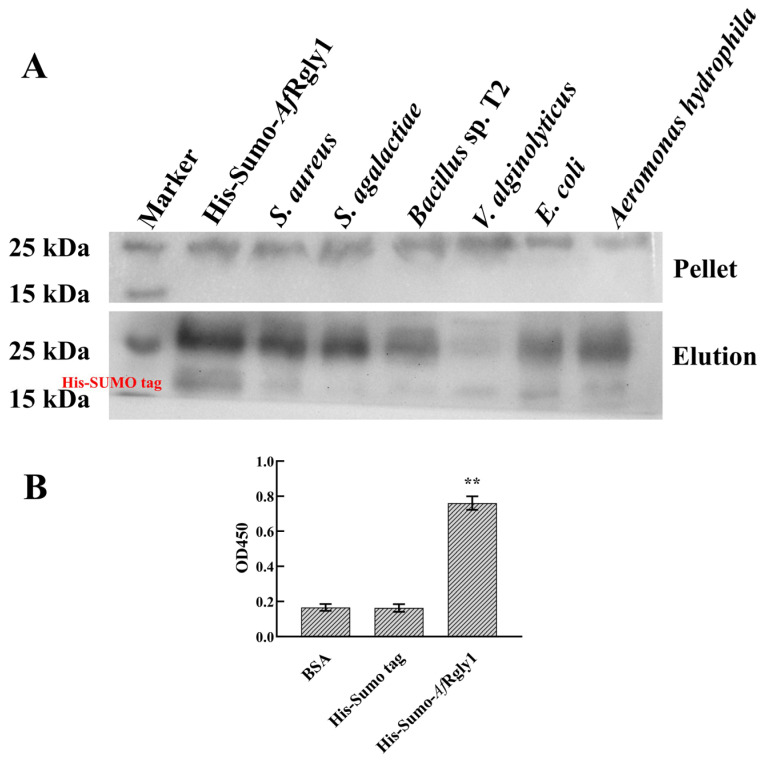
Microorganism-binding activity and membrane mimetic-binding activity of r*Af*Rgly1. (**A**) Microorganism-binding activity. His-SUMO-*Af*Rgly1 was detected by Western blot analysis after treatment with bacteria. His-SUMO-*Af*Rgly1 was used as a positive control. Upper panel, final pellet fractions; lower panel, elution fractions. (**B**) Membrane-mimicking binding activity assay. Bovine serum albumin (BSA) served as the control, while the His-SUMO tag acted as the negative control. The experiment was conducted with three biological repeats, each containing three technical replicates. **, significant difference compared to the control (BSA) at the level of 0.01.

The His-SUMO-*Af*Rgly1 containing His-SUMO tag was used for the microorganism-binding assay. The negative control His-SUMO tag cannot bind to bacteria. It was found that r*Af*Rgly1 binds to *S. Aureus*, *Bacillus* sp. T2, *S. agalactiae*, *A. hydrophila*, *E. coli*, and *V. alginolyticus*. These findings suggest that r*Af*Rgly1 may interact with bacterial cells (Figure 4A), contributing to its antibacterial activity. The ratio of the membrane mimics is consistent with the membrane components and proportions used in molecular dynamics. r*Af*Rgly1 can combine with the membrane mimics, which is in line with the results of molecular dynamics simulations (Figure 4B).

### 2.8. Effects of rAfRgly 1 on Membrane and Bacterial Morphology

**Figure 5:**
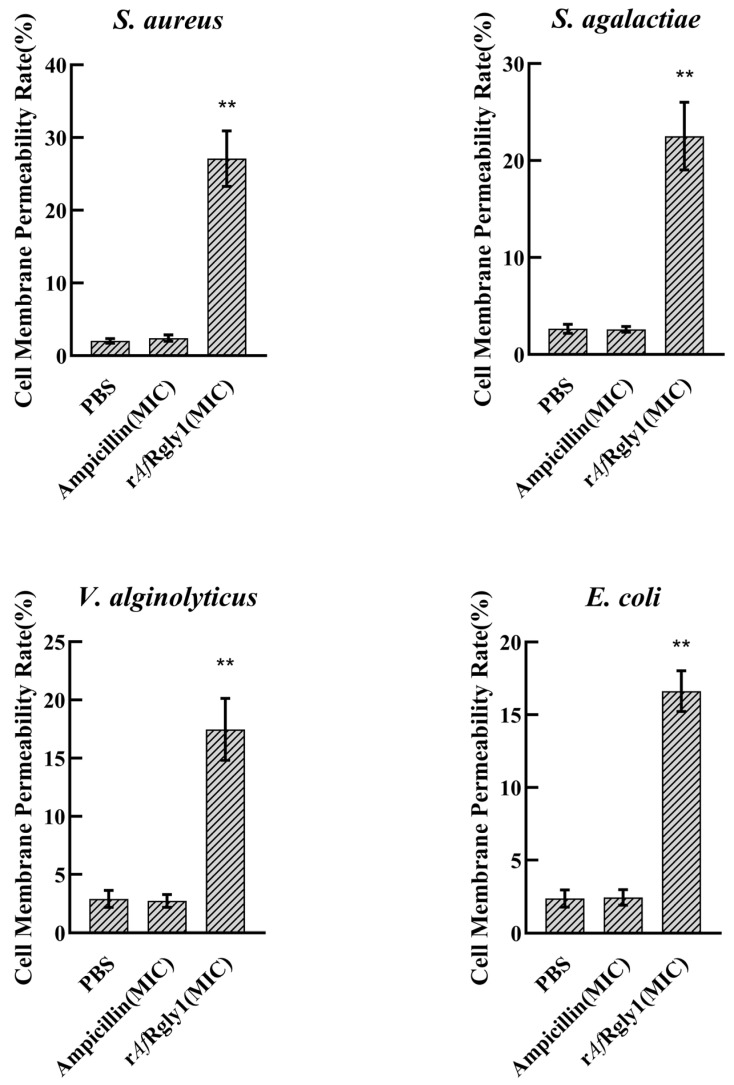
Effect of r*Af*Rgly1 on membrane permeability. Effect of r*Af*Rgly1 on the membrane permeability rate of *S. aureus*, *Bacillus* sp. T2, *V. alginolyticus* and *A. hydrophila*, respectively. These assays included three biological replicates, each with three technical replicates. **, significant difference compared to the control (PBS) at the level of 0.01.

Following the disruption of microbial membrane integrity, intracellular lactate dehydrogenase is released, which serves as an indicator of bacterial cell membrane permeability. After being treated by r*Af*Rgly1 at MICs for 2 h, the membrane permeability of *S. aureus*, *S. agalactiae*, *V. alginolyticus,* and *E. coli* was measured to be 27.11%, 22.51%, 17.47%, and 16.62%, respectively (Figure 5).

**Figure 6:**
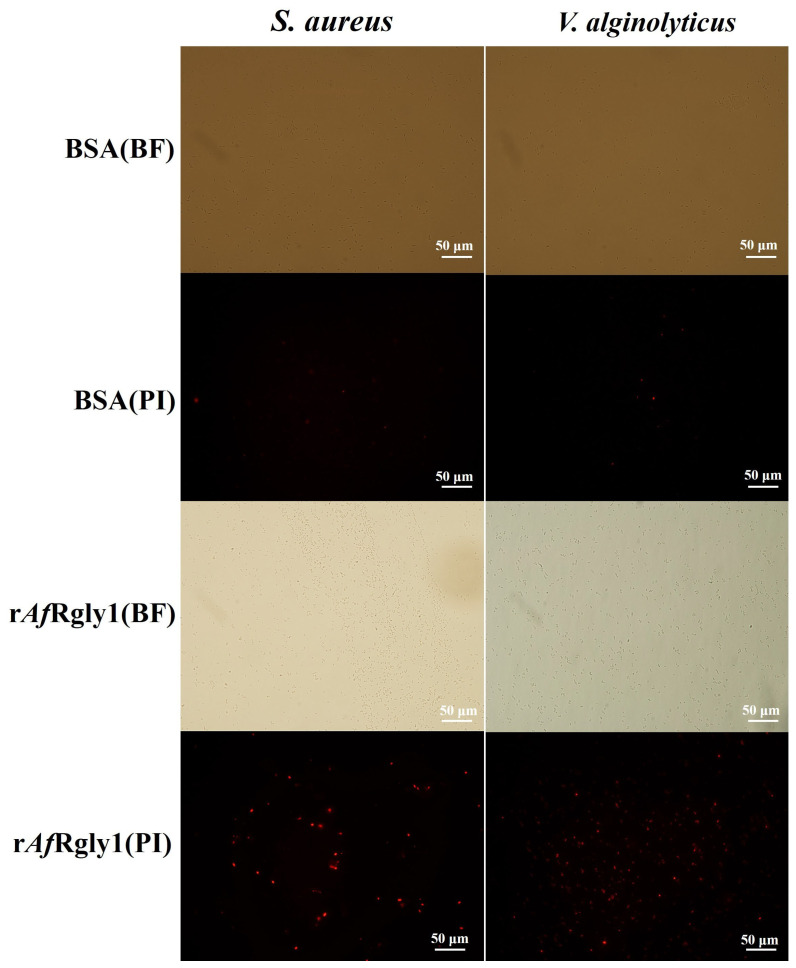
The effect of r*Af*Rgly1 on bacterial cell membrane integrity. About 1 × 10^6^ CFU·mL^−1^ bacteria were incubated with MBC of r*Af*Rgly1 for 2 h. PI: the cells were stained with PI and observed for PI uptake with a fluorescence microscope; BF: the bright field image. The scales are 50 μm.

Propidium iodide (PI) is unable to penetrate bacteria with intact cell membranes. It can selectively enter bacteria with compromised membrane integrity and subsequently bind to intracellular DNA. PI staining demonstrated that treatment with r*Af*Rgly1 led to substantial penetration of PI into bacterial cells in both *S. aureus* and *V. alginolyticus* (Figure 6), suggesting that r*Af*Rgly1 can cause destabilization of bacterial cell membranes.

**Figure 7:**
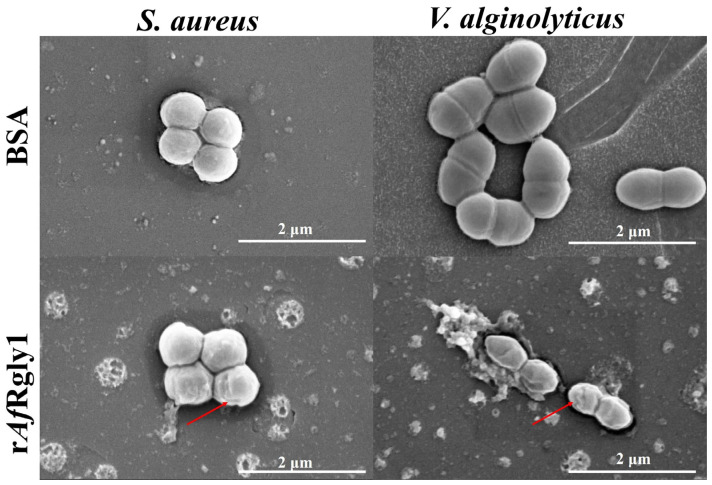
Morphological alterations in bacterial cells following r*Af*Rgly1 treatment. Bacterial samples (approximately 10^6^ CFU·mL^−1^) were exposed to the MBC of r*Af*Rgly1 for 2 h and examined using SEM. BSA served as the control group. The scale bars represent 2 μm. The red arrows denote regions where membrane contraction occurs.

Scanning electron microscope (SEM) observations revealed that S. aureus and V. alginolyticus displayed abnormal morphology features after exposure to rAfRgly1, including notable cellular shrinkage (Figure 7).

### 2.9. DNA-Binding Activity and Hemolytic Activity of rAfRgly

**Figure 8:**
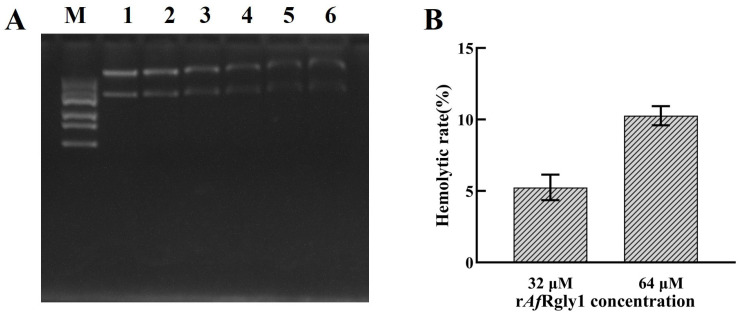
DNA-binding activity and hemolytic activity of r*Af*Rgly1. (**A**) Binding activity of r*Af*Rgly1 to plasmid DNA. Lane M, marker; lane 1, 64 μM BSA; lane 2–6, 4 μM, 8 μM, 16 μM, 32 μM, and 64 μM r*Af*Rgly1. (**B**) Hemolytic effect of r*Af*Rgly1 on red blood cells of fish. The hemolysis percentage was 0% in the 1× PBS (pH 7.4) group, whereas complete lysis (100%) was observed in the 0.2% Triton X-100-treated samples. Experiments were conducted with three biological repeats, each consisting of three technical repeats.

The SDS-PAGE results showed that after incubation with the plasmid for 2 h, 32 μM and 64 μM of r*Af*Rgly1 could significantly retard the migration of double-stranded DNA. At a concentration of 64 μM, the hemolysis rate of r*Af*Rgly on fish blood cells was 10.26%(Figure 8).

## 3. Discussion

The escalating crisis of antimicrobial resistance in aquaculture due to the indiscriminate use of antibiotics seriously threatens the sustainable development of the global aquaculture industry [25,26]. AMPs have been considered as ideal candidates to replace traditional antibiotics due to their unique bactericidal mechanism and low risk of drug resistance induction [27,28]. When attacked by pathogenic microorganisms, AMPs are promptly secreted through immune regulatory mechanisms [29,30]. Consistently, the expression levels of genes encoding AMPs are significantly up-regulated [29,30]. Screening AMPs based on transcriptome data serves as a reliable and efficient strategy during the identification of novel AMPs [31]. Moreover, AMPs frequently contain signal peptides that enable their activity outside the cellular environment [32,33]. Specifically, glycine-rich AMPs are typically characterized by a glycine content exceeding 20% [14,15,34]. Based on the transcriptomics data and the above characteristics, we discovered a candidate glycine-rich AMP and named it *Af*Rgly1.

*Af*Rgly1 demonstrates antimicrobial properties and effectively suppresses the growth of various bacterial species. Nevertheless, its antibacterial potency was shown to be less than that of *Pp*Rcys1 based on findings from our previous research [35]. AMPs like *Pp*Rcys1, which are rich in cysteine, can form stable β-sheet structures through intramolecular disulfide bonds [36,37]. With this rigid conformation, AMPs can effectively penetrate bacterial membranes [38,39]. *Af*Rgly1 contains three cysteine residues, which may theoretically form a disulfide bond. However, AlphaFold3 did not predict the formation of disulfide bonds in *Af*Rgly1. To further investigate the presence of disulfide bonds, circular dichroism spectroscopy will be employed in subsequent experiments. AlphaFold3 predicts that *Af*Rgly1 predominantly consists of a linear peptide (Figure 1D). Molecular dynamics simulations indicate that its structure is highly flexible (Figure 3), potentially contributing to its susceptibility to protease degradation [40]. This structural characteristic may partially explain why its activity is lower compared to *Pp*Rcys1. Although both *Af*Rgly1 and *Pp*Rcys1 target bacterial cell membranes, *Af*Rgly1 possesses the additional capability of DNA binding, a functional property absent in *Pp*Rcys1. Consequently, despite exhibiting a weaker antibacterial effect compared to *Pp*Rcys1, AfRgly1 remains a viable candidate as a template for antimicrobial peptide design.

The outer membrane of Gram-negative bacteria consists of lipopolysaccharides, phospholipids, and outer membrane proteins, of which the lipid A moiety of LPS is negatively charged and can attract cationic AMPs through electrostatic interactions [41,42]. However, the high-density lipopolysaccharides and lipoproteins of the outer membrane form a physical barrier that limits the penetration of AMPs [41,42]. In contrast to Gram-negative bacteria, Gram-positive bacteria have no outer membrane but a thick peptidoglycan layer [43,44]. Its negatively charged membrane components, such as teichoic acid, are more likely to bind directly to the cationic region of AMPs [43,44]. This may explain the better antibacterial activity of *Af*Rgly1 against Gram-positive bacteria than against Gram-negative bacteria.

The structural and physicochemical characteristics of *Af*Rgly1 provide important insights into its potential antimicrobial mechanisms and functional properties. With a molecular weight of 9.79 kDa and an isoelectric point of 9.80, *Af*Rgly1 exhibits strong cationic properties (net charge +9.25) that are typical features of many membrane-active AMPs, suggesting that *Af*Rgly1 is likely to interact with negatively charged bacterial membranes [27,33]. The near-neutral Grand Average Hydropathy (GRAVY) value of 0.001 indicates a balanced amphipathic nature, which is crucial for both membrane interaction and water solubility [45]. Therefore, molecular dynamics simulations combined with molecular experiments were conducted to assess its potential for membrane disruption.

The molecular dynamics simulation results reveal the dynamic process of *Af*Rgly1 approaching and interacting with the membrane. RMSD and molecular dynamics simulation snapshots (Figure 3A,D–F) indicate that the *Af*Rgly1 protein undergoes a significant conformational change before 200 ns and subsequently stabilizes to a relatively stable state, suggesting that *Af*Rgly1 requires an initial adaptation period to establish a stable interaction with the membrane. Linear peptides riching in glycine typically exhibit high structural flexibility, as evidenced by the changes in Rg values observed at 50 ns and 200 ns during the process of *Af*Rgly1 binding to the membrane (Figure 3B). These fluctuations suggest dynamic local unfolding and folding events. The sequential insertion pattern of positively charged lysine residues (LYS50 and LYS85 at 100 ns and extended to LYS37 and LYS45 at 300 ns, Figure 3E,G) indicates a time-dependent enhancement of protein–membrane interactions. Cationic residues usually play a key role in the binding of AMPs to bacterial membranes [46,47]. This is also confirmed by our previous molecular dynamics simulation results for *Pp*Rcys1 and *Pp*Crus-SWD1 [35,48].

To verify the binding ability of *Af*Rgly1 to bacterial membranes, membrane mimics identical to those used in the molecular dynamics simulations were constructed, and ELISA experiments were employed. The results demonstrate that *Af*Rgly1 is capable of binding to these membrane mimics, which corroborates the findings from the molecular dynamics simulations. Furthermore, Western blot analysis confirmed that *Af*Rgly1 interacts with intact bacteria, which is also corroborated by the ELISA results. These results indicate that *Af*Rgly1 can interact with bacterial cell membranes and has the potential to disrupt them. SEM revealed that the bacterial cell surface shrank after treatment with *Af*Rgly1. Additionally, PI staining and membrane permeability tests confirmed that *Af*Rgly1 altered the permeability of the bacterial cytoplasmic membrane. Hence, it can be concluded that the bacterial cell membrane is one of the targets of *Af*Rgly1.

Most AMPs carry positive charges, while the phosphate backbone of DNA is negatively charged; thus, they may non-specifically bind through electrostatic attraction. For instance, the linear isomer of melittin can wrap around DNA in a flexible conformation; similarly, the linear AMP indolicidin is disordered in solution, and its conformational flexibility may facilitate the binding to DNA [49]. Based on these findings, we hypothesized that DNA might be a potential intracellular target of *Af*Rgly1 in bacteria. To verify it, electrophoretic mobility shift assays were conducted. Results showed that when 32 μM of *Af*Rgly1 was co-incubated with plasmid DNA, the migration rate of the plasmid DNA in agarose gel was significantly decreased, indicating the existence of binding of plasmid DNA with *Af*Rgly1. Therefore, bacterial DNA may be another target of *Af*Rgly1.

In vitro hemolysis testing, as outlined by Ketan Amin and Rose-Marie Dannenfelser in their guidance for pharmaceutical scientists, indicates that formulations with a hemolysis value of less than 10% are considered non-hemolytic, whereas values exceeding 25% are deemed to pose a potential risk for hemolysis [50]. In this study, the hemolysis rate of r*Af*Rgly1 at 64 μM was 10.26%. Therefore, we will add cell experiments in the subsequent period to evaluate its biological toxicity. The membrane activity characteristics of recombinant peptides, especially those related to cell membranes, may be directly attributed to the presence of uncleared signal peptides in their structure [51]. Therefore, the presence of signal peptides may be one of the factors leading to the hemolysis rate of *Af*Rgly1 for FBCs exceeding 10% at 64 μM. The further research direction is to optimize *Af*Rgly1 to reduce its hemolytic toxicity and enhance its antibacterial activity.

The N-terminal region of *Af*Rgly1 contains a signal peptide, and its N-terminal segment is incomplete. When organisms secrete antimicrobial peptides, they usually remove their signaling peptides. But signal peptides may therefore represent a validated target for drug design [52], and thus were retained. We employed an 18 kDa His-SUMO tag and fused it with *Af*Rgly1, starting from SER1, to facilitate protein expression. The use of a larger His-SUMO tag may provide steric protection to the potentially unstable N-terminal region, thereby preserving the native N-terminal structure and preventing possible cleavage by signal peptidases [53]. And the His-SUMO fusion system was used to produce AMPs in *E. coli* BL21(DE3) and can reduce its toxicity to the host [54,55]. By optimizing the induction conditions (such as reducing the IPTG concentration) [49,56], we successfully decreased the unit yield of the AMP and ultimately obtained recombinant *Af*Rgly1 (r*Af*Rgly1). But these operations contribute to the structural differences between r*Af*Rgly1 and its natural counterpart, *Af*Rgly1, which may result in variations in their antibacterial activities. In the future, we plan to construct a mutant of r*Af*Rgly1 that lacks the signal peptide in order to obtain a structure closer to its native form. Subsequently, the antibacterial activity of this mutant will be assessed to evaluate the influence of the signal peptide on the antibacterial properties of *Af*Rgly1.

Recombinant antimicrobial peptides may exhibit reduced antibacterial activity compared to their natural counterparts when expressed in the *E. coli* system, primarily due to the limited capacity of this system for post-translational modifications, such as incomplete or absent glycosylation, phosphorylation and disulfide bond formation [57]. Recombinant *Af*Rgly1 without undergoing appropriate oxidation and renaturation processes is unlikely to form correct disulfide bonds [57]. In contrast, naturally occurring *Af*Rgly1 is expected to possess a defined disulfide bond structure. Therefore, differences in antibacterial activity may exist between r*Af*Rgly1 and native *Af*Rgly1 [38,57]. To further investigate this, we plan to extract native *Af*Rgly1 and determine its disulfide bond connectivity using techniques such as mass spectrometry or diagonal electrophoresis. Concurrently, oxidative-reductive treatments will be applied to r*Af*Rgly1 to facilitate proper disulfide bond formation. Ultimately, the functional impact of disulfide bonds will be assessed by comparing the antibacterial activities of native *Af*Rgly1, r*Af*Rgly1, and refolded r*Af*Rgly1.

The inherent limitations of LC-MS technology in the analysis of antimicrobial peptides include reduced detection sensitivity for hydrophilic or low-molecular-weight peptides, such as those containing continuous glycine residues or polar regions, which is attributed to poor ionization efficiency and weak chromatographic retention [58]. Furthermore, the application of trypsin in this study to cleave lysine or arginine residues may hinder the precise identification of certain peptides [59]. It is likely that these factors contributed to the LC-MS identification results covering only 38.14% of the complete peptide sequence of *Af*Rgly1.

## 4. Materials and Methods

### 4.1. Bacterial Strains and Culture Conditions

The experimental bacteria included three Gram-positive strains—*S. aureus* (ATCC 6538), *S. agalactiae* (ATCC 51487), and *Bacillus* sp. T2—as well as six Gram-negative strains—*Aeromonas hydrophila* (ATCC 35654), *V. alginolyticus* (ATCC 17749), *V. anguillarum* (ATCC 14181), *E. coli* (ATCC 8739), *V. harveyi* (ATCC 43516), and *Acinetobacter* sp. L32, which were kindly provided by Professor Chaogang Wang and Xiaohui Cai [60,61]. Bacteria were initially preserved as glycerol stocks and then cryopreserved at –80 °C for long-term storage [62]. Prior to experimentation, the bacterial stock (50 μL) was thawed in 2 mL of growth medium and incubated at 37 °C with shaking at 200 rpm for 12 h to ensure their revival and proper activation. Specifically, *S. aureus*, *Bacillus* sp. T2, *Acinetobacter* sp. L32, *E. coli,* and *A. hydrophila* were cultured in Luria–Bertani (LB) medium (ST163, Beyotime, Shanghai, China), while *V. harveyi, V. alginolyticus,* and *V. anguillarum* were propagated in Zobell Marine Agar 2216E (HB0132, Haibo, Qingdao, China).

### 4.2. Quantitative Analysis of AfRglys Expression

Sixty *A. franciscana*, originally collected from the Penglai Mozhikou Marine Aquaculture Facility in Penglai, China, were acclimatized in sterile seawater supplemented with ampicillin (100 μg/mL) for 48 h. This study employed the double parallel experimental design, dividing *A. franciscana* into a pathogen-stressed treatment group (*n* = 2) and a PBS-treated control group (*n* = 2). Each group consisted of 15 individuals of *A. franciscana*, which were treated with 10^6^ CFU/mL pathogen suspension and an equal volume of sterile PBS, respectively. After 6 h, four groups of *A. franciscana* were immediately frozen in liquid nitrogen. Total RNA was extracted from these four samples simultaneously using the Trizol method (KL-0016, Shanghai Kanglang Biology Technology Co., Ltd., Shanghai, China). After digestion with DNase I, the RNA quality was strictly checked, with the requirement that the OD260/280 ratio detected by Nanodrop was between 1.8 and 2.0 and the RIN value detected by Agilent 2100 was ≥7.0. Consequently, the Hieff NGS^®^ ds-cDNA Synthesis Kit (13488ES08, Yease, Shanghai, China) was used to construct cDNA libraries for these four samples simultaneously, and PE150 sequencing was performed on the NovaSeq 6000 platform. During the data analysis, Hisat2 (T2.2.1)was used for sequence alignment [63] and StringTie was used for transcript assembly [64]. The coefficient of gene expression between the two groups of parallel samples was required to be >0.9. Differential expression analysis was conducted using the edge R package (3.3.5)(*p* value < 0.05) [65].

### 4.3. Prediction and Identification of Glycine-Rich AMP

The genomic data of *A. franciscana* was obtained from the NCBI database (accession: GCA_032884065.1). Using a screening pipeline, we selected sequences meeting the following criteria: length < 100 amino acids, glycine content > 20%, subcellular localization outside the cell, and having a signal peptide. In addition, the expression level should be significantly upregulated after stimulation by pathogenic microorganisms. Signal peptide prediction was performed using SignalP 6.0 [66], whereas WoLF PSORT (https://wolfpsort.hgc.jp/, accessed on 14 August 2025) was used to determine subcellular localization [67]. Structural modeling of the homologous protein *Af*Rgly1 was performed using AlphaFold3 [68], while physicochemical properties were predicted via APD3 (http://aps.unmc.edu/AP/ (accessed on 1 January 2025)). A search for AMPs with sequence similarity to *Af*Rgly1 was conducted using the CAMPR3 database [69].

### 4.4. Expression and Purification of Recombinant AfRgly1

The codon-optimized *Af*Rgly1 (NCBI: KAK2718072.1) according to the *E. coli* preference was fused to the His-SUMO tag and expressed in *E. coli* BL21(DE3). The synthetic 348 bp gene (with additional BamHI/XhoI sites) was cloned into the pSmartI vector (5850 bp; Supplementary Figure S1) and transformed into competent cells. Positive clones (kanamycin-resistant) were verified by PCR/sequencing using general primers of pSmartI (EF:5′-TTA AGA TTC TTG TAC GAC GG-3′, ER: 5′-TGC TAG TTA TTG CTC AGC GG-3′).

Established positive clones were then inoculated into LB medium supplemented with kanamycin (100 μg/mL) and incubated overnight at 37 °C with shaking at 200 rpm. On the following day, the overnight bacterial culture was then inoculated into fresh LB medium using a 1:100 dilution ratio. The suspension was subsequently incubated at 37 °C with continuous shaking at 200 rpm until reaching mid-log phase (OD_600_ ≈ 0.6). Protein expression was induced by the addition of isopropyl-β-d-thiogalactopyranoside (IPTG), which was adjusted to a final concentration of 0.2 mM, followed by a 12 h induction period at 37 °C.

Bacterial cell collection and lysis were conducted as previously described [48]. The cells were disrupted using the TieChui *E. coli* lysis buffer, followed by centrifugation at 4 °C for 30 min at a speed of 10,000 rpm to isolate the supernatant from the cellular debris. Crude proteins were extracted from uninduced and induced cells and analyzed via 12% SDS-PAGE. The recombinant His-SUMO-*Af*Rgly1 peptide was then purified from the clarified cell lysate using nickel-affinity column chromatography. Post-purification, the target protein was subjected to buffer exchange dialysis against 1× PBS for 24 h at 4 °C. The His-SUMO tag was then completely cleaved with an additional 1 U of SUMO protease (General Biosystems, Chuzhou, China) and incubated overnight at 4 °C. The reaction mixture was then passed through the column again, with the cleaved His-SUMO tags being bound to the Ni-NTA resin, allowing tag-free r*Af*Rgly1 to be collected in the flow-through. Purified protein samples were then subjected to SDS-PAGE analysis for quality assessment, with protein quantification performed using a BCA-based assay kit (Beyotime) according to standard protocols. Obtained protein solutions were divided into aliquots, freeze-dried, and preserved at −80 °C in powdered form. To confirm the identity, integrity, and purity of the obtained recombinant protein, r*Af*Rgly1 samples were analyzed by Wininnovate Bio Company (Shenzhen, China) using liquid chromatography–mass spectrometry (LC-MS) analysis.

### 4.5. Antimicrobial Activity Determination

The antimicrobial efficacy of recombinant *Af*Rgly1 (r*Af*Rgly1) was assessed employing a microdilution technique based on the CLSI-recommended protocol. Bacterial suspensions with an OD600 of 0.4 were standardized to a concentration of 10^4^ CFU/mL in Mueller–Hinton broth [37]. Two-fold serial dilutions of rAfRgly1, ranging from 64 to 1 μM in PBS, were prepared in 96-well microtiter plates, with each well containing 20 μL of peptide solution and 80 μL of bacterial inoculum. Phosphate-buffered saline (PBS) was used as the negative control. Following an 18 h incubation at 37 °C, microbial growth was monitored by measuring optical density at 560 and 590 nm (OD560 and OD590). The minimum inhibitory concentration (MIC) was defined as the lowest peptide concentration that prevented visible color change, as detected by the resazurin-based viability indicator.

To further investigate the growth inhibition kinetics, bacterial growth curves were generated by measuring OD_600_ at time intervals of 0, 4, 8, 12, and 24 h under MIC and half-MIC (0.5× MIC) conditions. Bovine serum albumin (BSA) was used as the control protein. The minimum bactericidal concentration (MBC) was determined through a colony-counting assay. After 18 h of incubation, 10 μL aliquots from wells showing no visible growth (sample OD600 ≤ control OD600) were transferred onto Mueller–Hinton agar (MHA, HB0128, Haibo, Qingdao, China) plates and incubated at 37 °C for 24 h. The MBC was identified as the lowest peptide concentration resulting in ≥99.9% bacterial reduction, confirmed by the absence of colony formation. Ampicillin and recombinant PpRcys1 (rPpRcys1) were used as positive controls, while 1× PBS served as the negative control.

All experiments were performed in triplicate to ensure reproducibility and data consistency [35].

### 4.6. Molecular Dynamics (MD) Simulations Analysis

To investigate the interaction between *Af*Rgly1 and bacterial membranes, a series of molecular dynamics (MD) simulations was conducted, emphasizing the significance of peptide-membrane binding in computational models. The CHARMM36 force field was employed to describe the structural and dynamic properties of *Af*Rgly1 [70]. The membrane system was constructed using 324 POPE lipids and 162 POPG lipids per leaflet, based on previously established membrane compositions [71,72]. Solvation was achieved using the TIP3P water model [73]. A cubic simulation box with dimensions of 12 × 12 × 12 nm^3^ was set up for systems. To ensure electrostatic neutrality, appropriate numbers of Na^+^ and Cl^−^ ions were introduced into the system. All molecular dynamics simulations were performed using GROMACS version 2023.3 [74]. VMD 1.9.3 [75] was used for trajectory visualization.

Non-bonded interactions were calculated with a cutoff distance of 1.2 nm for both the Lennard–Jones and short-range electrostatic interactions. For long-range electrostatic interactions, the particle mesh Ewald (PME) method was applied, with a grid spacing of 0.16 nm and a fourth-order spline interpolation [76,77]. Temperature regulation was achieved using the V-rescale thermostat at 310 K, which is above the phase transition temperature of POPG, with a coupling time constant of 1.0 ps [78]. Pressure was maintained semi-isotropically at 1 bar using the C-rescale method, with a time constant of 5 ps and a compressibility of 4.5 × 10^−5^ (kJ·mol^−1^·nm^−3^)^−1^ [79]. Bond constraints were applied using the LINCS algorithm [80], allowing for a stable integration time step of 2 fs. Prior to production runs, the system underwent energy minimization, followed by a 500 ps equilibration phase under both NVT (constant number of particles, volume, and temperature) and NPT (constant number of particles, pressure, and temperature) conditions. Finally, a 500 ns production simulation was conducted to analyze the behavior of *Af*Rgly1.

### 4.7. Electron Microscopy

Electron microscopy analysis was performed as established protocols with modifications [81]. Mid-log phase cultures of *S. aureus* and *V. alginolyticus* were collected and standardized to 10^6^ CFU/mL using PBS. Bacterial suspensions were treated with r*Af*Rgly1 at MBC level on coverslips (24-well plate, 2 h incubation), with BSA-treated samples as controls. Post-treatment specimens underwent sequential processing: primary fixation in 5% glutaraldehyde (4 °C, 10 h), PBS rinses (3×), gradient ethanol dehydration (30–100%, 10 min/step at 4 °C), critical point drying (Hitachi-HCP system, Hitachi, Tokyo, Japan), and gold sputter-coating (Hitachi MC1000, Hitachi, Tokyo, Japan). Imaging was performed using a Thermo Fisher APREO S SEM(Thermo Fisher, Waltham, MA, USA).

### 4.8. Microorganism-Binding Assay

Western blot analysis was employed to evaluate the r*Af*Rgly1-bacterial binding ability. Briefly, bacterial suspensions at a concentration of 1 × 10^8^ CFU/mL were incubated with 200 μL of a mixed solution containing His-SUMO-*Af*Rgly1 and His-SUMO tag, where each component was present at a concentration of 10 μM, under rotary agitation at room temperature for 1 h. Washed thrice with TBS, the microbial pellets and supernatants were separated by centrifugation (10,000 rpm for 5 min) and then subjected to SDS-PAGE analysis. His-SUMO-*Af*Rgly1 and His-SUMO tag served as positive/negative controls, respectively. Polyvinylidene fluoride (PVDF) membranes were blocked with 5% skim milk/TBST and then probed with HRP-anti-His antibody (Boyi Biotech, Boyi, China, 1:30,000). Protein bands were visualized using BeyoECL Plus chemiluminescent substrate (Beyotime, Shanghai, China) and chemiluminescent detection system (WD-9423B/C, Liuyi, Beijing, China) following standard protocols, and triplicate biological replicates were performed.

### 4.9. Binding Assay for Membrane Mimetic

To determine the optimal lipid composition, a molar ratio of POPE to POPG (7.5 nmol: 2.5 nmol) was selected within a total lipid concentration of 100 μM. The lipid mixture was initially dissolved in chloroform, followed by solvent evaporation under a nitrogen stream and further drying under high vacuum for 1 h to ensure complete removal of residual organic solvent. Liposome preparation was initiated by rehydrating the dried lipid film with preheated HEPES buffer (20 mM HEPES, 150 mM NaCl, pH 7.4). The resulting suspension underwent vortex mixing and sonication—either through pulsed probe sonication on ice (10 s bursts for 10 cycles) or bath sonication at 55 °C for 30 min—to generate small unilamellar vesicles.

The prepared liposomes were diluted to a working concentration of 10–20 μg/mL and added to a 96-well microplate at 100 μL per well. The plate was incubated overnight at 4 °C to allow liposome adsorption onto the well surface. Following this, each well was rinsed three times with 1× phosphate-buffered saline containing Tween 20 (PBST, pH 7.4). To block nonspecific binding sites, 100 μL of 5% skim milk in 1× PBST (pH 7.4) was added to each well, and the plate was incubated at 37 °C for 2 h. Afterward, the wells were gently washed three times with 1× PBST (pH 7.4) to remove excess blocking agent.

His-SUMO-*Af*Rgly1 was prepared at a concentration of 10 μM in 1× PBS (pH 7.4) and added to the respective wells. For comparative analysis, 10 μM bovine serum albumin (BSA) served as the positive control, while 10 μM His-SUMO tag alone was used as the negative control. The plate was incubated at 37 °C for 1 h, followed by a single wash with 1× PBST (pH 7.4). Subsequently, 100 μL of horseradish peroxidase (HRP)-conjugated anti-His antibody, diluted 1:5000 in 1× PBST (pH 7.4) (Boyi, Changzhou, China), was added to each well. The plate was then incubated at 37 °C for another hour, followed by five washes with 1× PBST (pH 7.4) to remove unbound antibody.

To detect binding, 100 μL of TMB substrate solution was added to each well to initiate color development. The reaction was terminated immediately by adding 200 μL of ELISA stop solution per well. Absorbance at 450 nm was recorded using a microplate reader (SynergyTM LX, BioTek, Kaysville, UT, USA). To ensure experimental reliability, the assay was performed with three biological replicates and three technical replicates.

### 4.10. Lactate Dehydrogenase (LDH) Release Assay

The membrane-disruption activity of *Af*Rgly1 against *S. aureus*, *S. agalactiae*, *V. alginolyticus*, and *E. coli* was assessed through an LDH release assay. In brief, bacterial cultures in the mid-logarithmic growth phase (OD_600_ ≈ 0.5) were harvested, rinsed, and resuspended in phosphate-buffered saline (PBS). Aliquots of 100 μL of the bacterial suspension were then exposed to *Af*Rgly1 at its minimum inhibitory concentration (MIC) in a 96-well plate format for 2 h. Following centrifugation at 12,000× *g* for 2 min, 50 μL of the supernatant was combined with 50 μL of reaction solution containing 50 mM sodium phosphate buffer (pH 7.5), 0.6 mM pyruvate, and 0.2 mM NADH, and the mixture was allowed to react at ambient temperature for 10 min. The reaction was terminated by adding 50 μL of 1 M acetic acid, and the absorbance was recorded at 340 nm. To determine total LDH content, bacterial cells lysed with 1% Triton X-100 were used as a reference [82,83]. The percentage of LDH released was subsequently calculated as follows:

$$Permeability\left(\%\right)=\frac{OD340\left(Sample\right)-OD340(BSA)}{OD340\left(TritonX-100\right)-OD340(BSA)}\times 100\%$$

with statistical significance assessed by one-way ANOVA (triplicate, *p* < 0.05). Controls included bacteria treated by BSA (negative) and 1% Triton X-100 (positive).

### 4.11. PI Staining

*S. aureus* and *V. alginolyticus* were cultured as described above at their MBC for 2 h. Samples were stained with a PI staining kit (Sangon, Shanghai, China) according to the manufacturer’s instructions. Cells were observed under a fluorescence microscope (BX51, Olympus, Tokyo, Japan).

### 4.12. DNA-Binding Assay

The *V. parahaemolyticus* PirA virulence gene (GenBank: MH410659.1) was synthesized with additional *BamHI*/*XhoI* flanking sites and cloned into the pSmartI vector to construct pSmart-PirA (Supplementary Figure S2). DNA–protein binding reactions (20 μL) containing 400 ng plasmid DNA and serially diluted r*Af*Rgly1 (16–64 μM) in Tris-based buffer (10 mM of Tris-HCl, pH of 8.0, 1 mM of EDTA, 20 mM of KCl, 1 mM of DTT, 5% glycerol, 50 μg/mL of BSA) were incubated at 37 °C for 1 h. BSA at 64 μM was used as the negative control. Samples mixed with loading buffer were electrophoresed on 1% agarose gel at 120 V for 20 min. The DNA retardation patterns were used to indicate binding activity.

### 4.13. Hemolytic Activity Assay

The hemolytic potential of recombinant proteins was evaluated using carp fish erythrocytes. Fish red blood cells (FRBCs, HQ80080, Hongqian Bio, Guangzhou, China) were washed with sterile saline (0.85%) and resuspended to a final 4% (*v*/*v*) suspension. Aliquots (150 μL) were incubated with equal volumes of test protein (16–64 μM), PBS (the negative control), or 0.2% Triton X-100 (the positive control) at 37 °C for 1 h. After centrifugation, supernatant absorbance at 570 nm (OD_570_) was measured. Hemolysis percentage was calculated as

$$Hemolysis\left(\%\right)=\frac{OD570\left(TritonX-100\right)-OD570\left(PBS\right)}{OD570\left(sample\right)-OD570\left(PBS\right)}\times 100\%$$

Triplicate biological and technical replicates were performed to ensure data reliability.

### 4.14. Statistical Analysis

Statistical analysis was conducted using GraphPad Prism 10.0 (GraphPad, San Diego, CA, USA). Significance was assessed with a one-way analysis of variance (ANOVA), and all data were reported as mean ± SD from three biological replicates. *p*-value < 0.05 was deemed to be statistically significant.

## 5. Conclusions

In this study, a novel AMP, *Af*Rgly1, was successfully identified and hetero-expressed, according to the features of glycine-rich AMPs and transcriptomic analysis of brine shrimp. *Af*Rgly1 exhibits broad-spectrum antibacterial activity and is capable of exerting its effects by compromising the integrity of bacterial cell membranes. Furthermore, it may also interact with bacterial DNA. Within the effective antibacterial concentration range, *Af*Rgly1 exhibits low selectivity for fish red blood cells. These findings indicate that *Af*Rgly1 has substantial potential for pharmaceutical development.

## Figures and Tables

**Table 1:** Minimal inhibitory concentrations (MICs) and minimum bactericidal concentration (MBC)of r*Af*Rgly1 against Gram-positive and Gram-negative bacteria.

Microorganism		MIC			MBC
		r*Af*Rgly1	r*Pp*Rcys1	Ampicillin	r*Af*Rgly1
Gram^+^	*S. aureus*	32	8	2	128
	*Bacillus* sp. T2	32	8	-	128
	*S. agalactiae*	64	16	8	128
Gram^−^	*A. hydrophila*	64	32	-	256
	*Acinetobacter* sp. L32	-	32	-	--
	*E. coli*	64	16	64	128
	*V. alginolyticus*	64	16	64	256
	*V. harveyi*	-	32	-	--
	*V. anguillarum*	64	32	-	256

“-” indicates that the compound did not exhibit inhibitory activity at a concentration of 64 μM. “--” indicates that the compound did not exhibit bactericidal activity at a concentration of 256 μM.

## Data Availability

All data generated or analyzed during this study are included in this published article, and further inquiries can be directed to the corresponding authors.

## Supplementary Materials

The following supporting information can be downloaded at: https://www.mdpi.com/article/10.3390/md23080330/s1, Figure S1: pSmartI-*Af*Rgly1 Plasmid map; Figure S2: pSmart-PirA Plasmid map; Figure S3: original Western blot; Table S1: screening of rich glycine peptides from *Artemia franciscana* genome and transcriptome; Table S2: results of transcriptome gene differential expression analysis. Table S3: the comparison result of AfRgly1 in the CAMPR3 database.

## Abbreviations

The following abbreviations are used in this manuscript:

AMP	antimicrobial peptide
Rg	gyration
RMSD	root mean square distances
RMSF	root mean square fluctuations
POPE	1-palmitoyl-2-oleoyl-sn-glycero-3-phosphoethanolamine
POPG	phosphatidylglycerol
