# Supplementary material for: Heterologous Expression and Antimicrobial Targets of a Novel Glycine-Rich Antimicrobial Peptide from Artemia franciscana
Source: Mar Drugs. 2025 Aug 17;23(8):330. doi: 10.3390/md23080330 (PMC12387859; doi:10.3390/md23080330)
Supplement: Supplementary file 1 [file marinedrugs-23-00330-s001.zip › Supplementary Table S1.pdf]

Supplementary table S1: Screening of rich glycine peptides from *Artemia franciscana* genome and transcriptome

Peptides	Gene ID	Glycine content	log_2FC	P value
KAK2701352.1	QYM36_019995	28.26%	-0.16115	1
KAK2702693.1	QYM36_018700	26.32%	0	1
KAK2702937.1	QYM36_018467	37.23%	-8.90689	0.125019
KAK2704045.1	QYM36_017621	23.23%	0	1
KAK2708149.1	QYM36_013912	20.99%	0	1
KAK2710398.1	QYM36_011796	20.99%	0	1
KAK2710400.1	QYM36_011798	25.61%	0	1
KAK2710401.1	QYM36_011799	23.17%	0	1
KAK2712709.1	QYM36_011408	32.61%	0	1
KAK2713088.1	QYM36_011697	43.94%	0	1
KAK2718072.1(*Af*Rly1)	QYM36_006760	21.65%	1.319819469	6.38E-32
KAK2719619.1	QYM36_005183	30.85%	0	1
KAK2719799.1	QYM36_005315	23.17%	0	1
KAK2724339.1	QYM36_000998	26.04%	0	1
KAK2725568.1	QYM36_000161	21.28%	0	1
KAK2726099.1	QYM36_000529	21.33%	0	1
