# Supplementary material for: Heterologous Expression and Antimicrobial Targets of a Novel Glycine-Rich Antimicrobial Peptide from Artemia franciscana
Source: Mar Drugs. 2025 Aug 17;23(8):330. doi: 10.3390/md23080330 (PMC12387859; doi:10.3390/md23080330)
Supplement: Supplementary file 1 [file marinedrugs-23-00330-s001.zip › Supplementary Tabls S3.pdf]

Supplementary Table S3. The comparison result of AfRgly1 in the CAMPR3 database.

Sequences ID in CAMPR3	Score	E Value
CAMPSQ322(Holotricin-3)	38.9	2e-006
CAMPSQ1503(Bacteriocin microcin B17)	37.4	4e-006
CAMPSQ3913	37.7	8e-006
CAMPSQ3914	37.7	8e-006
CAMPSQ1508	37.4	1e-005
