# Supplementary figures and images for: Heterologous Expression and Antimicrobial Targets of a Novel Glycine-Rich Antimicrobial Peptide from Artemia franciscana
Source: Mar Drugs. 2025 Aug 17;23(8):330. doi: 10.3390/md23080330 (PMC12387859; doi:10.3390/md23080330)

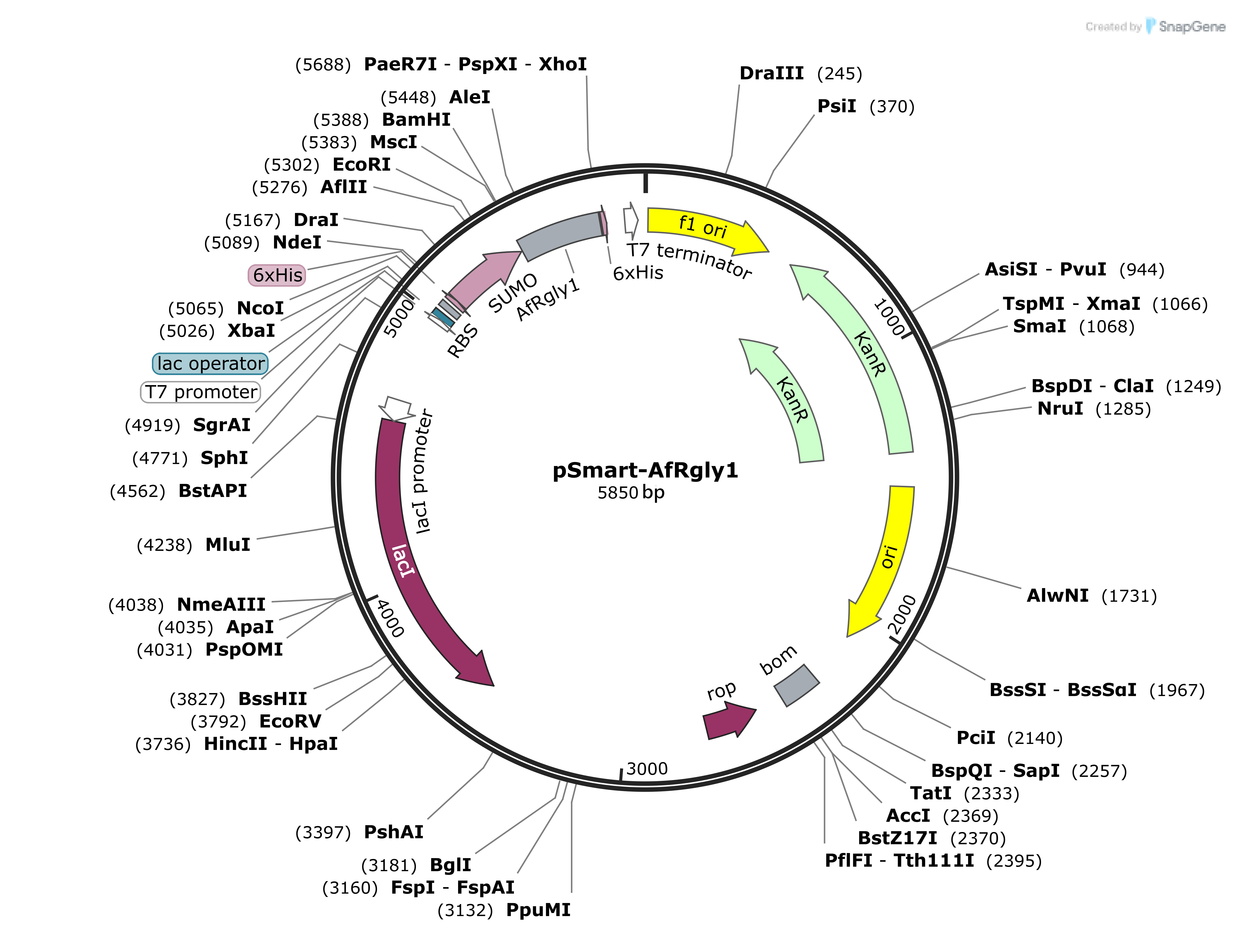

Supplement: Supplementary file 1 [file marinedrugs-23-00330-s001.zip › Supplementary Figure S1.tif]

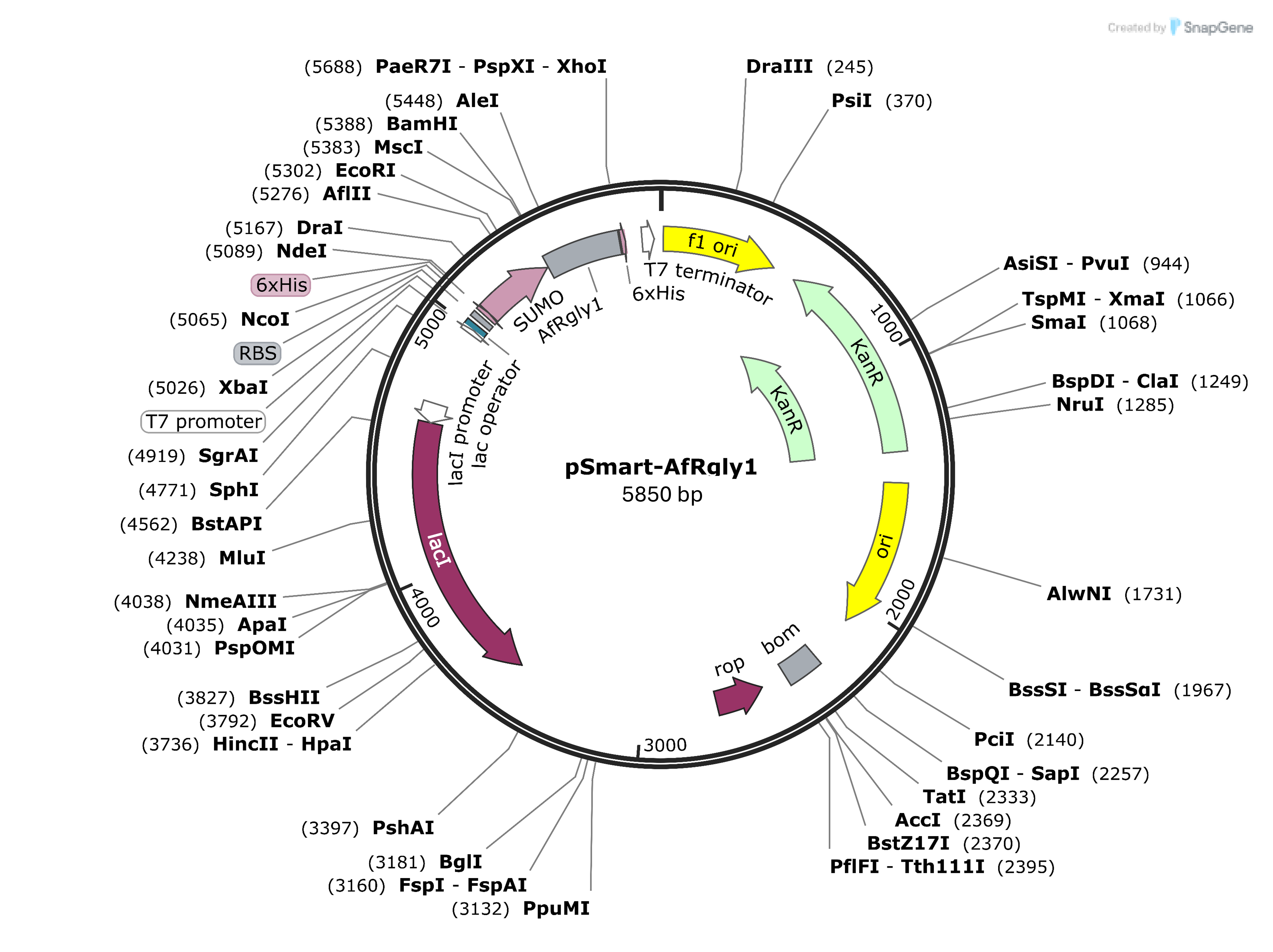

Supplement: Supplementary file 1 [file marinedrugs-23-00330-s001.zip › Supplementary Figure S2.tif]

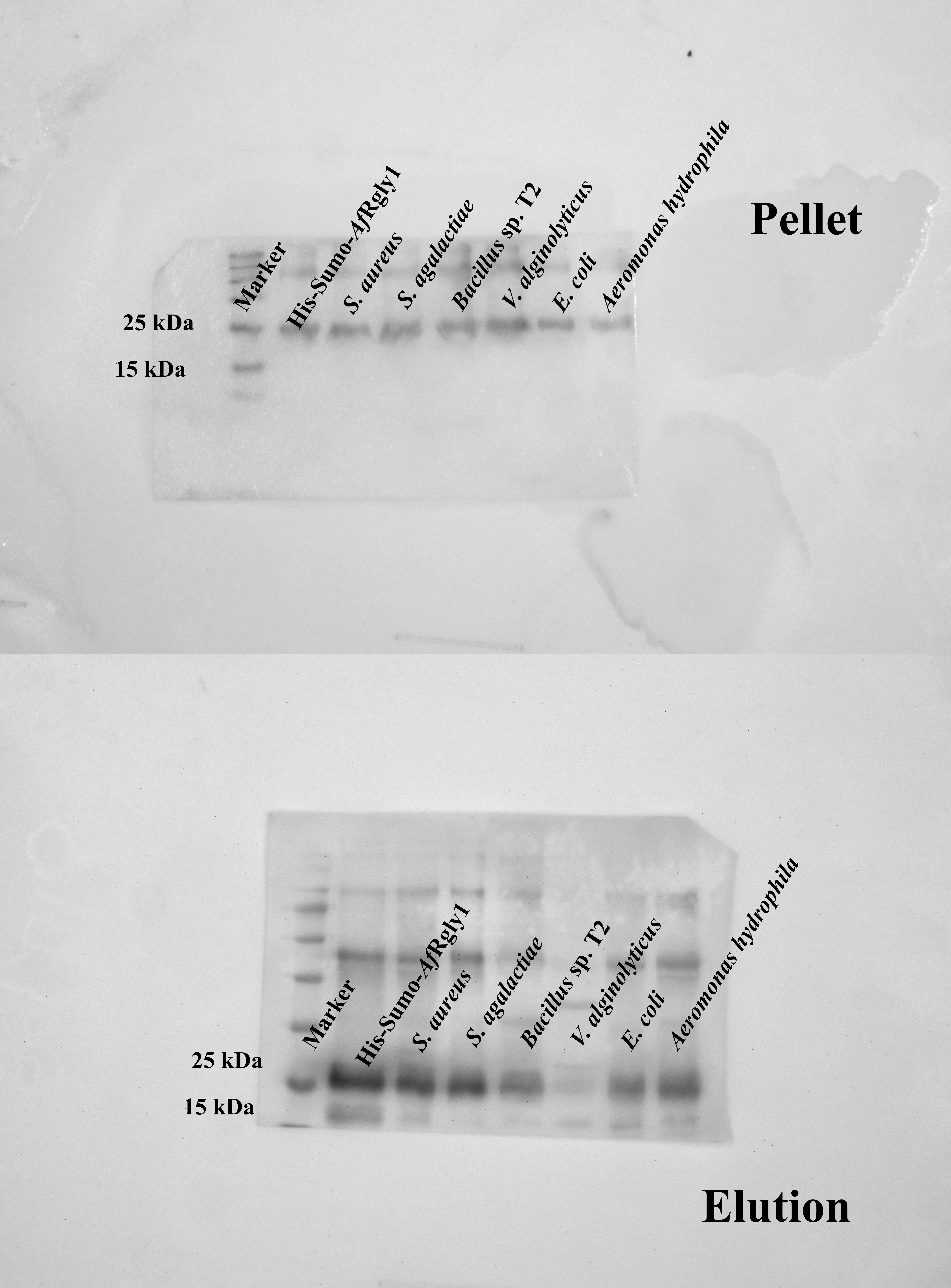

Supplement: Supplementary file 1 [file marinedrugs-23-00330-s001.zip › Supplementary Figure S3-wb origin.jpg]
